# Improving the diagnostic performance for prior COVID-19 with T-SPOT, an interferon-gamma release assay

**DOI:** 10.3389/fmicb.2025.1675605

**Published:** 2025-09-24

**Authors:** Masaki Yamamoto, Michael T. White, Takahisa Kawaguchi, Kazuya Setoh, Yusuke Tsuda, Koh Shinohara, Yasuhiro Tsuchido, Yasufumi Matsumura, Fumihiko Matsuda, Miki Nagao

**Affiliations:** ^1^Department of Clinical Laboratory Medicine, Graduate School of Medicine, Kyoto University, Kyoto, Japan; ^2^Infectious Disease Epidemiology and Analytics G5 Unit, Department of Global Health, Institut Pasteur, Université Paris-Cité, INSERM U1347, Paris, France; ^3^Department of Human Disease Genomics, Center for Genomic Medicine, Graduate School of Medicine, Kyoto University, Kyoto, Japan; ^4^Department of Epidemiology for Community Health and Medicine, Kyoto Prefectural University of Medicine, Kyoto, Japan; ^5^Kyoto-Pasteur International Collaborative Research Unit for Vaccinomics, Graduate School of Medicine, Kyoto University, Kyoto, Japan

**Keywords:** COVID-19, diagnosis, antibody test, IGRA, T-SPOT

## Abstract

**Introduction:**

The precise diagnosis of a prior COVID-19 infection remains challenging. This study aimed to evaluate the efficacy of T-SPOT assays for diagnosing prior SARS-CoV-2 infections by using frozen peripheral blood mononuclear cells (PBMCs) combined with antibody tests.

**Methods:**

The study included 122 participants with PCR-confirmed COVID-19 (the positive control cohort) and 67 participants with no evidence of prior infection (the negative control cohort). Antibody testing was conducted using iFlash-SARS-CoV-2 IgG (YHLO, iF_N) and MAGPIX^®^ assays (Luminex, Lumi_N), which target the nucleocapsid protein. T-SPOT^®^ Discovery SARS-CoV-2 assays (Oxford Immunotec) were used to detect cell-mediated immune responses against nucleocapsid (Tspot_N) and membrane (Tspot_M) proteins.

**Results:**

Antibody tests had similar sensitivities (if_N: 67.2% and Lumi_N: 64.8%) and specificities (>98.4%). The Tspot_N assay demonstrated comparable performance to the antibody tests, with a sensitivity, specificity, and area under the receiver operating characteristic curve (AUC) of 62.5% (95% confidence interval: 52.0%–72.2%), 98.4% (95% CI: 91.2%–100.0%), and 0.923, respectively. The Tspot_M assay had lower sensitivity (15.3%). The combination of the Tspot_N test and the Lumi_N antibody test significantly improved the sensitivity and AUC to 88.0% and 0.979, respectively (*p* = 0.012). Net reclassification improvement and integrated discrimination improvement analyses further supported the improved diagnostic performance of the combination assay.

**Conclusion:**

Frozen PBMCs were useful for performing T-SPOT assays. The combination of T-SPOT assays targeting nucleocapsid protein and antibody tests improved the diagnosis of past SARS-CoV-2 infections in vaccinated participants. These findings suggest that integrating cellular and humoral immunity assays can facilitate COVID-19 prevalence studies.

## 1 Introduction

Conducting prevalence studies through serosurveillance (antibody testing) is essential for comprehensively understanding national and regional outbreaks and were particularly important during the early phase of the COVID-19 pandemic (Public Health Collaborators on Serosurveillance for Pandemic Preparedness and Response PHSeroPPR, 2023). In case–control studies, such as those comparing COVID-19 patients with a non-infected cohort, precise confirmation of cases is crucial. Direct viral detection methods, such as real-time RT-PCR and viral antigen tests, are highly effective for identifying infections. However, these methods may not be useful for detecting asymptomatic cases because individuals without symptoms are less likely to undergo diagnostic testing (Kronbichler et al., 2020). Antibody tests are technically capable of detecting prior SARS-CoV-2 infections, as they can reflect the humoral immune response against specific antigens, with detectable levels emerging approximately 2 weeks post infection onset. Despite their utility, however, antibody tests exhibit low sensitivity during the initial stages of COVID-19, and it is well documented that antibody titers against SARS-CoV-2 gradually decline over time (Aiello et al., 2022). Thus, antibody tests have limited efficacy in providing evidence of past SARS-CoV-2 infection.

The interferon-gamma (IFN-γ) release assay (IGRA) is an alternative infection detection method that, although used primarily to diagnose tuberculosis, can also reflect cellular immunity from prior SARS-CoV-2 infections (Pai et al., 2014). The IGRA quantifies IFN-γ released from T cells following stimulation with antigenic proteins. In the context of SARS-CoV-2, IFN-γ release is predominantly measured with three methods: (i) an enzyme-linked immunosorbent assay (ELISA), (ii) an enzyme-linked immunosorbent spot (ELISPOT) assay, and (iii) flow cytometry (FCM) (Johnson et al., 2023, Törnell et al., 2022). Several studies have highlighted the greater positivity of IGRAs compared with antibody tests as a significant advantage (Murugesan et al., 2022, Törnell et al., 2022). Therefore, IGRAs, when utilized in conjunction with antibody tests, are expected to play a complementary role in the diagnosis of COVID-19. Given the global dissemination of COVID-19 vaccines, particularly mRNA vaccines that establish immunity against SARS-CoV-2 spike proteins, seroprevalence assessments should target proteins other than the spike protein, such as the nucleocapsid (N) and membrane proteins (Hayden et al., 2024). Although the cell-mediated immune responses against SARS-CoV-2 spike proteins have been extensively evaluated, assessments of responses against the nucleocapsid or membrane protein remain limited in the literature (Törnell et al., 2022, Mak et al., 2024, Liu et al., 2021).

Consequently, to evaluate the accuracy in diagnosing past COVID-19 infections, we assessed the performance of two categories of testing assay, antibody tests and IGRAs, as well as their combinations, that detect immune responses against the nucleocapsid and/or membrane proteins.

## 2 Materials and methods

### 2.1 Study design and participants

In this study, 309 individuals, primarily hospitality workers in the city of Kyoto, were initially recruited. A high prevalence of prior COVID-19 was noted among these individuals, for whom sample collection involved both blood collection and administration of a questionnaire. The study was conducted from August to September 2022, coinciding with the seventh pandemic wave and the prevalence of BA.5 Omicron strain. All vaccines administered to the participants in this study (Pfizer, Moderna, and Novavax) targeted the original SARS-CoV-2 strain. Details of the vaccination status are presented in Supplementary Table 1.

**FIGURE 1:**
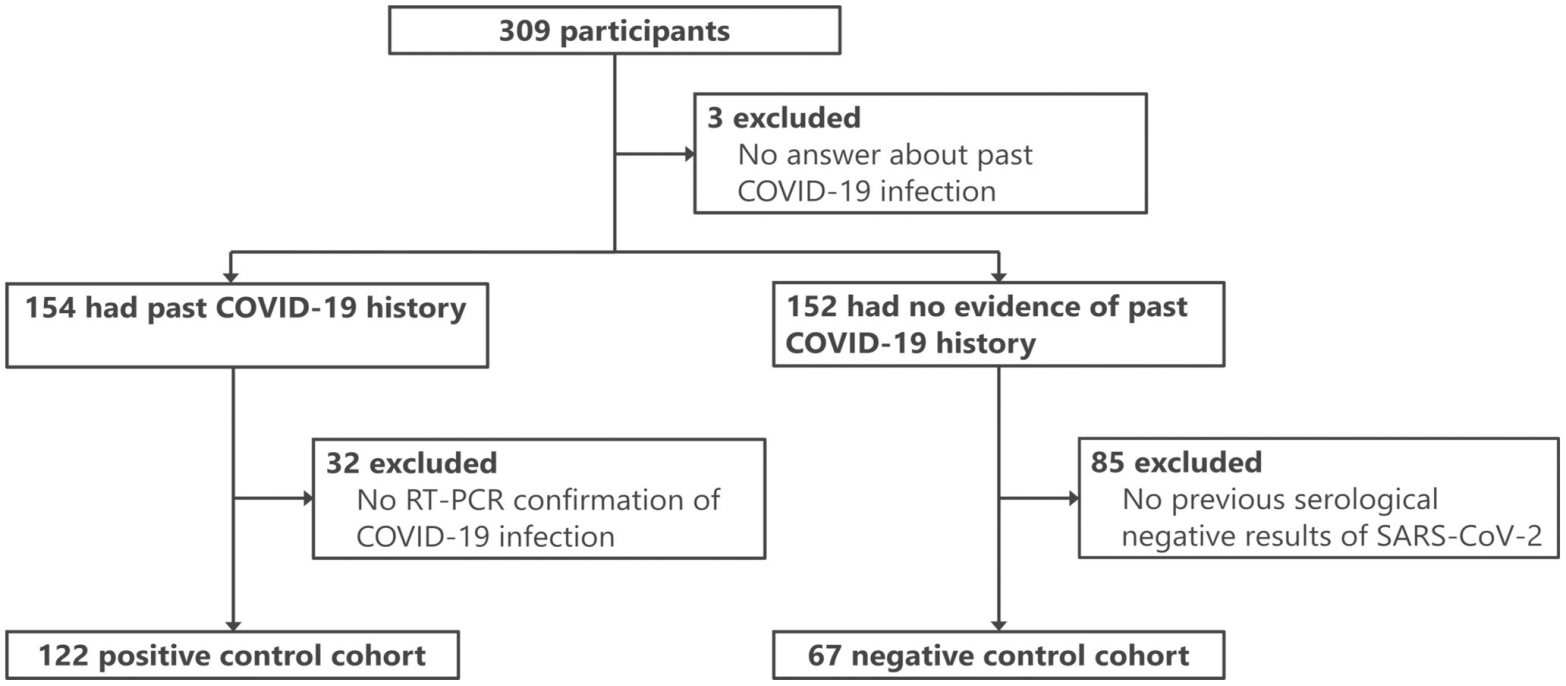
Flowchart of the participant profiles.

For this study, 189 participants were selected from the 309 initially recruited individuals (Figure 1) and divided into two cohorts. The positive control (PC) cohort consisted of 122 participants with RT-PCR-confirmed COVID-19 (either current or prior). The PCR methodology adhered to the standards set by the National Institute of Infectious Diseases, Japan (Shirato et al., 2021), and targeted the N gene of SARS-CoV-2. These tests were conducted in clinical laboratories. The negative control (NC) cohort, meanwhile, comprised 67 individuals with no evidence of COVID-19 according to the questionnaire and three negative antibody test results. To further substantiate the absence of SARS-CoV-2 infection in the NC cohort, the iFlash-SARS-CoV-2 IgG assay (YHLO, Shenzhen, China) was performed on two available serum samples collected in November 2020 and between February and March 2021, confirming negativity for the disease (see details below). The characteristics of the two cohorts are summarized in Table 1.

**TABLE 1:** Baseline characteristics of the participants.

Cohort	Total (*n* = 309)	PC (*n* = 122)	NC (*n* = 67)	PC + NC (*n* = 189)	*P*-value (PC vs. NC)
**Sex**					0.420
Male (*n*, %)	40 (12.9%)	12 (9.8%)	10 (14.9%)	22 (11.6%)	
Female (*n*, %)	269 (87.1%)	110 (90.2%)	57 (85.1%)	167 (88.4%)	
**Age (median, IQR)**	49.5 (27–65.25)	36 (22–55)	66 (55–74.5)	51 (27.75–67.5)	**<0.001**
**Age category (*n*, %)**					
<= 20	46 (14.9%)	21 (17.2%)	1 (1.5%)	22 (11.6%)	
21–30	43 (13.9%)	33 (27.0%)	0 (0.0%)	33 (17.5%)	
31–40	29 (9.4%)	13 (10.7%)	5 (7.5%)	18 (9.5%)	
41–50	39 (12.6%)	14 (11.5%)	5 (7.5%)	19 (10.1%)	
51–60	56 (18.1%)	17 (13.9%)	16 (23.9%)	33 (17.5%)	
61–70	29 (9.4%)	9 (7.4%)	8 (11.9%)	17 (9.0%)	
71–80	53 (17.2%)	12 (9.8%)	27 (40.3%)	39 (20.6%)	
81–90	11 (3.6%)	2 (1.6%)	5 (7.5%)	7 (3.7%)	
91–100	2 (0.6%)	0 (0.0%)	0 (0.0%)	0 (0.0%)	
No answer	1 (0.3%)	1 (0.8%)	0 (0.0%)	1 (0.5%)
**Occupation category (*n*, %)**					**<0.001**
Hospitality_worker	238 (77.0%)	111 (91.0%)	44 (65.7%)	155 (82.0%)	
Office_worker	37 (12.0%)	7 (5.7%)	12 (17.9%)	19 (10.1%)	
Others	18 (5.8%)	1 (0.8%)	5 (7.5%)	6 (3.2%)	
No occupation	1 (0.3%)	0 (0.0%)	0 (0.0%)	0 (0.0%)	
No answer	15 (4.9%)	3 (2.5%)	6 (9.0%)	9 (4.8%)	
**Vaccination**	294 (95.1%)	114 (93.4%)	65 (97.0%)	179 (94.7%)	0.499
Pfizer	169 (57.5%)	68 (59.6%)	41 (63.1%)	109 (60.9%)	
Pfizer and moderna	57 (19.4%)	18 (15.8%)	11 (16.9%)	29 (16.2%)	
Moderna	36 (12.2%)	16 (14.0%)	4 (6.2%)	20 (11.2%)	
Pfizer and novavax	1 (0.3%)	1 (0.9%)	0 (0.0%)	1 (0.6%)	
No answer	31 (10.5%)	11 (9.6%)	9 (13.8%)	20 (11.2%)	
Valid_count* (*n*, %)	301 (97.4%)	120 (98.4%)	63 (94.0%)	183 (96.8%)	0.188

PC, positive control; NC, negative control; IQR, interquartile range. Bold values denote *p*-values less than 0.05. *This number indicates that T-SPOT^®^ Discovery results can be obtained.

### 2.2 Blood samples

Serum and peripheral blood mononuclear cells (PBMCs) were obtained from each participant. Serum samples were collected in blood collection tubes containing serum separation gel (Terumo, Tokyo, Japan). The serum was separated by centrifugation at 1500 × *g* for 10 min and subsequently stored at −80 °C until use. For PBMC separation, blood samples were collected in tubes containing sodium heparin as the anticoagulant (Terumo). In accordance with the manufacturer’s instructions, a Leucosep tube with separation medium (Greiner Bio-One, Kremsmünster, Austria) was used for PBMC separation. The PBMCs were then preserved in CELLBANKER1 (TaKaRa Bio, Shiga, Japan), which ensures a viability rate exceeding 70% over 1 year at −80 °C. Each stored sample was thawed immediately prior to use.

### 2.3 Antibody tests

Antibody testing was conducted using two instruments to ensure robustness of the results. First, antibody tests using the iFlash3000 instrument (iF_N assay), specifically the iFlash-SARS-CoV-2 IgG assay (YHLO), were performed within 1 week of blood collection. This semiquantitative method uses multiple chemiluminescent immunoassays with the nucleocapsid protein as the primary antigen. The assay cutoff value was established at 10 AU/mL, at which a previous study reported a sensitivity of 94.5% (95% confidence interval: 91.7%–96.6%) and a specificity of 100% (95% CI: 96.4%, 100%) (Yamamoto et al., 2022). Additionally, the MAGPIX^®^ system (Luminex, Texas, USA) (Lumi_N assay) was used to obtain quantitative antibody results, in accordance with the methodology documented by Rosado et al. (2021) 1 year after blood collection. The analysis employed the median fluorescence intensity (MFI) with a cutoff value of 1908 MFI, as determined in this study, ensuring a target specificity greater than 99%. The nucleocapsid proteins used included SARS-CoV-2 NPv1, obtained from Institut Pasteur and SARS-CoV-2 NPv2, obtained from Native Antigen (REC31812-100).

### 2.4 Interferon-gamma (IFN-γ) release assay

An IGRA, specifically the T-SPOT^®^ Discovery SARS-CoV-2 (Oxford Immunotec, Oxfordshire, United Kingdom), was conducted 1–7 months after blood collection. This assay consists of four panels to detect immune responses against the SARS-CoV-2 spike protein (panel 1), nucleocapsid protein (panel 3), membrane protein (panel 4), and proteins from endemic strains of coronaviruses (panel 13). During the study period, the majority of participants received at least one course of vaccination targeting the SARS-CoV-2 spike protein. Panel 1 (spike protein) was excluded from this study because the results cannot be used to distinguish between participants with a history of COVID-19 and those who were vaccinated. Consequently, in this study, the responses to non-vaccine target proteins, such as the SARS-CoV-2 nucleocapsid (Tspot_N assay) and membrane (Tspot_M assay) proteins, were evaluated. Viable PBMCs were counted using trypan blue staining with Cell Counter model R1 (Olympus, Tokyo, Japan). According to the manufacturer’s instructions, 2.5 × 10^5^ cells/well were used for T-SPOT^®^ Discovery analysis. Upon completion of the T-SPOT assays, spot-forming cells were counted using an ImmunoSpot S6 Entry M2 Analyzer (Cellular Technology Ltd., Ohio, USA), followed by visual confirmation of the results. PBMC preparations for T-SPOT^®^ Discovery SARS-CoV-2 PBMCs were counted using Cell Counter model R1 (Olympus, Tokyo, Japan), which can distinguish between viable and dead cells. Viable PBMCs were prepared at a final concentration of 2.5 × 10^5^ cells/100 μL for the T-SPOT^®^ Discovery assay, in accordance with the manufacturer’s instructions. The T-SPOT assay results were classified in accordance with the manufacturer’s guidelines: positive (spot count ≥ 8), borderline (5–7), negative (≤4), and indeterminate (negative control well > 10 or positive control well < 20). These classifications were employed to evaluate the positivity rate, sensitivity, specificity, positive predictive value (PPV), negative predictive value (NPV), and McNemar’s test. For additional statistical analyses, spot counts were used to treat T-SPOT assays as quantitative measures. In the combination assays, a positive result was determined if at least one of the assays yielded a positive outcome, whereas a negative result was determined only if all assays yielded negative outcomes.

### 2.5 Statistical analysis

All the statistical analyses were performed using R version 4.4.1 (R Foundation for Statistical Computing, Vienna, Austria). Statistical methods and libraries (packages) were employed as follows. The chi-square test or Fisher’s exact test was used for comparing categorical variables between groups. The Mann-Whitney U test was used to compare continuous variables between groups. The sensitivity, specificity, PPV, and NPV were calculated with the epiR library, incorporating information on the duration between blood collection and infection or vaccination. Receiver operating characteristic (ROC) curve analysis was performed to determine the area under the curve (AUC) and assess the performance of each assay. McNemar’s test was used to compare the sensitivities and specificities between assays. The agreement rate and Cohen’s kappa (κ) coefficient were calculated for all assays in the irr library to determine the level of agreement and concordance, respectively, between assays. The PredictABEL library was used to calculate the net reclassification improvement (NRI) and integrated discrimination improvement (IDI) to assess the efficacy of the combination assay (Pencina et al., 2008). The two-category NRI and the continuous NRI were employed for NRI evaluation (Pencina et al., 2017), with the reclassification cutoff set to the Youden index. Additionally, the ΔAUC and Brier scores were calculated to further evaluate the efficacy of the combination assay (Hilden and Gerds, 2014).

## 3 Results

### 3.1 Summary of the results of each assay

The results of the T-SPOT assay are presented in Supplementary Table 2. Notably, approximately 20% of the PC cohort and 8%–10% of the NC cohort exhibited borderline or indeterminate results, which were subsequently excluded from further analysis.

The positivity rates for each assay are listed in Supplementary Table 3. The two antibody tests (iF_N and Lumi_N) and Tspot_N assays had similar positivity rates. In quantitative assays, the median values of the PC cohort were significantly greater than those of the NC cohort. Seven discrepancies were found among the antibody tests (five results for iF_N-positive/Lumin_N-negative and two results for iF_N-negative/Lumi_N-positive). However, no discrepancy was found in the NC cohort. The Tspot_M assay had the lowest the positivity rate of all assays. Thirty-six discrepancies were observed among the T-SPOT assays (34 results for Tspot_N-positive/Tspot_M-negative, and two results for Tspot_N-negative/Tspot_M-positive). False-positive results were found in 1.6% of Tspot_N and 3.2% of Tspot_M assays. The interval between the last recorded infection and the subsequent blood collection in the PC cohort is shown in Supplementary Figure 1. The date of the most recent COVID-19 infection was not available for three out of 122 individuals in the PC cohort. Approximately half of the PC cohort (60/119 [50.4%]) experienced infection within 180 days of blood collection (Supplementary Table 4). Regarding the antibody test assays (iF_N and Lumi_N), the positivity rate in the PC cohort was significantly higher within 180 days postinfection than beyond 180 days postinfection (*p* < 0.001 for each antibody test assay). Such differences were not observed in the T-SPOT assays (Tspot_N and Tspot_M), however. More than 90% of the participants had received vaccinations. The interval between the last vaccination and blood collection is also shown in Supplementary Figure 2.

In the PC cohort, 22 individuals with prior COVID-19 were asymptomatic (18.0%) (Supplementary Table 5). The positivity rate of the Tspot_N assay among symptomatic individuals was greater than that among asymptomatic individuals, although this difference was not statistically significant (*p* = 0.289). In the other assays, no significant differences in the positivity rate were observed between symptomatic and asymptomatic individuals.

### 3.2 Agreement rate and Cohen’s kappa (κ) concordance

The concordance rate between the antibody tests (iF_N and Lumi_N assays) was notably high (96.3%); however, the concordance rates between the antibody tests and T-SPOT assays were relatively low, ranging from 63.1% to 74.9% (Table 2). Cohen’s κ concordance analysis corroborated this trend; the κ value for the antibody tests was 0.924 (95% CI: 0.869–0.979), indicating almost perfect agreement. Conversely, the κ values between the antibody tests and T-SPOT assays ranged from 0.104 to 0.395, indicating no to slight-to-moderate agreement between the tests. The observed relationships between the antibody tests and T-SPOT assays, including similar sensitivity, specificity, AUC, and reclassification improvement values but lower concordance rates or κ values, imply differences in the detectable population contingent on the assay employed. These assays may improve performance when utilized in a complementary manner.

**TABLE 2:** Agreement rate and concordance between antibody tests and interferon-γ release assays.

Assays comparison	*N* *	Cohen’s kappa coefficient		Agreement
		Value (95% CI)	*P*-value	rate (%)
iF_N vs. Lumi_N	189	0.924 (0.869, 0.979)	**<0.001**	96.3
iF_N vs. Tspot_N	157	0.395 (0.248, 0.542)	**<0.001**	71.3
iF_N vs. Tspot_M	160	0.104 (-0.017, 0.224)	0.072	63.1
Lumi_N vs. Tspot_N	157	0.364 (0.215, 0.514)	**<0.001**	70.1
Lumi_N vs. Tspot_M	160	0.082 (−0.04, 0.204)	0.164	63.1
Lumi_N vs. Tspot_N & Tspot_M	155	0.392 (0.245, 0.539)	**<0.001**	71.0

CI, confidence interval; Lumi_N, nucleoprotein assay of the MAGPIX^®^ system (Luminex); iF_N, iFlash-SARS-CoV-2 IgG assay; Tspot_N, nucleocapsid assay of T-SPOT^®^ Discovery SARS-CoV-2; Tspot_M, membrane protein assay of T-SPOT^®^ Discovery SARS-CoV-2.

*Number excluding missing values. Bold values indicate a *p*-value of less than 0.05.

### 3.3 Sensitivity, specificity, positive predictive value (PPV), and negative predictive value (NPV)

The sensitivity of each assay ranged from 60% to 70%, whereas the specificity exceeded 96%, as determined by the default cutoff values described in the Section “2 Materials and methods” (Table 3). Compared with the other assays, the Tspot_M assay demonstrated the lowest specificity (15.3%). The PPVs and NPVs of the assays are presented in Table 3. Both antibody assays (iF_N and Lumi_N) exhibited equivalent performances across all metrics, with no significant difference in sensitivity according to McNemar’s test (*p* = 0.257) (Table 4). No difference was observed between the antibody assays and the Tspot_N assay in terms of specificity or sensitivity; however, the sensitivity of the Tspot_M assay was significantly lower than that of the antibody assays (*p* < 0.001). In Supplementary Table 6, the sensitivity and specificity metrics are detailed for each interval subsequent to the latest COVID-19 infection.

**TABLE 3:** Sensitivity, specificity, PPV, and NPV of each assay, including combination assays.

Assays	Sensitivity (95% CI)	Specificity (95% CI)	PPV (95% CI)	NPV (95% CI)
Lumi_N	64.8% (55.6%, 73.2%)	100.0% (94.6%, 100.0%)	100.0% (95.4%, 100.0%)	60.9% (51.1%, 70.1%)
iF_N	67.2% (58.1%, 75.4%)	100.0% (94.6%, 100.0%)	100.0% (95.6%, 100.0%)	62.6% (52.7%, 71.8%)
Tspot_N	62.5% (52.0%, 72.2%)	98.4% (91.2%, 100.0%)	98.4% (91.2%, 100.0%)	62.5% (52.0%, 72.2%)
Tspot_M*	15.3% (8.8%, 24.0%)	96.8% (88.8%, 99.6%)	88.2% (63.6%, 98.5%)	42.0% (33.8%, 50.5%)
Tspot_N* & Tspot_M*	67.0% (56.6%, 76.4%)	96.7% (88.7%, 99.6%)	96.9% (89.3%, 99.6%)	65.6% (54.8%, 75.3%)
Lumi_N* & Tspot_N*	88.0% (80.7%, 93.3%)	98.4% (91.2%, 100.0%)	99.0% (94.8%, 100.0%)	81.1% (70.3%, 89.3%)
Lumi_N & Tspot_N* & Tspot_M*	89.1% (82.0%, 94.1%)	96.8% (89.0%, 99.6%)	98.1% (93.5%, 99.8%)	82.4% (71.8%, 90.3%)
Lumi_N_yc	97.5% (93.0%, 99.5%)	80.6% (69.1%, 89.2%)	90.2% (83.7%, 94.7%)	94.7% (85.4%, 98.9%)
Tspot_N_yc	90.0% (83.2%, 94.7%)	84.1% (72.7%, 92.1%)	91.5% (85.0%, 95.9%)	81.5% (70.0%, 90.1%)
Tspot_M_yc	54.2% (44.8%, 63.3%)	85.7% (74.6%, 93.3%)	87.8% (78.2%, 94.3%)	49.5% (39.8%, 59.3%)
Tspot_N_yc & Tspot_M_yc	91.7% (85.2%, 95.9%)	74.6% (62.1%, 84.7%)	87.3% (80.2%, 92.6%)	82.5% (70.1%, 91.3%)
Lumi_N_yc & Tspot_N_yc	98.4% (94.2%, 99.8%)	71.4% (58.7%, 82.1%)	87.0% (80.2%, 92.1%)	95.7% (85.5%, 99.5%)
Lumi_N_yc & Tspot_N_yc & Tspot_M_yc	95.1% (89.6%, 98.2%)	76.1% (64.1%, 85.7%)	87.9% (81.1%, 92.9%)	89.5% (78.5%, 96.0%)

CI, confidence interval; PPV, positive predictive value; NPV, negative predictive value; Tspot_N, T-SPOT^®^ Discovery using nucleocapsid protein as stimulants; Tspot_M, T-SPOT^®^ Discovery using membrane protein as stimulants; iF_N, iFlash-SARS-CoV-2 IgG assay using iFlash 3000 instruments (YHLO); Lumi_N, antibody test targeting nucleocapsid protein using the MAGPIX^®^ system (Luminex). Each assay name with “yc” denotes the use of the Youden index to determine the cutoff (Lumi_N, 316; Tspot_N, 2; and Tspot_M, 2, respectively).

*Positive cutoff value is 8, according to the manufacturer’s guidelines.

**TABLE 4:** Comparative analysis of assay sensitivities and specificities with McNemar’s test.

Comparing assays, *p*-value of McNemar’s test	Sensitivity (95% CI)	Specificity (95% CI)	*N* *
**iF_N vs. Lumi_N**			189
iF_N	67.2% (58.1%, 75.4%)	100.0% (94.6%, 100.0%)	
Lumi_N	64.8% (55.6%, 73.2%)	100.0% (94.6%, 100.0%)	
*P*-vlaue	0.257	NA	
**iF_N vs. Tspot_N**			157
iF_N	62.5% (52.0%, 72.2%)	100.0% (94.1%, 100.0%)	
Tspot_N	62.5% (52.0%, 72.2%)	98.4% (91.2%, 100.0%)	
*P*-value	1.000	0.317	
**iF_N vs. Tspot_M**			160
iF_N	63.3% (52.9%, 72.8%)	100.0% (94.6%, 100.0%)	
Tspot_M	15.3% (8.8%, 24.0%)	96.8% (88.8%, 99.6%)	
*P*-value	<0.001	0.157	
**Lumi_N vs. Tspot_N**			157
Lumi_N	60.4% (49.9%, 70.3%)	100.0% (94.1%, 100.0%)	
Tspot_N	62.5% (52.0%, 72.2%)	98.4% (91.2%, 100.0%)	
*P*-value	0.768	0.317	
**Lumi_N vs. Tspot_M**			160
Lumi_N	61.2% (50.8%, 70.9%)	100.0% (94.2%, 100.0%)	
Tspot_M	15.3% (8.8%, 24.0%)	96.8% (88.8%, 99.6%)	
*P*-value	<0.001	0.157	
**Lumi_N vs. Tspot_N & Tspot_M**			155
Lumi_N	59.6% (49.0%, 69.6%)	100.0% (94.1%, 100.0%)	
Tspot_N & Tspot_M	67.0% (56.6%, 76.4%)	96.7% (88.7%, 99.6%)	

CI, confidence interval; iF_N, iFlash-SARS-CoV-2 IgG assay using iFlash 3000 instruments (YHLO); Lumi_N, antibody test targeting nucleocapsid protein using the MAGPIX^®^ system (Luminex); Tspot_N, T-SPOT^®^ Discovery using nucleocapsid protein as stimulants; Tspot_M, T-SPOT^®^ Discovery using membrane protein as stimulants; NA, not available.

*Number excluding missing values.

### 3.4 ROC curve analysis, AUC, DeLong’s test, and reclassification analysis (NRI and IDI)

**FIGURE 2:**
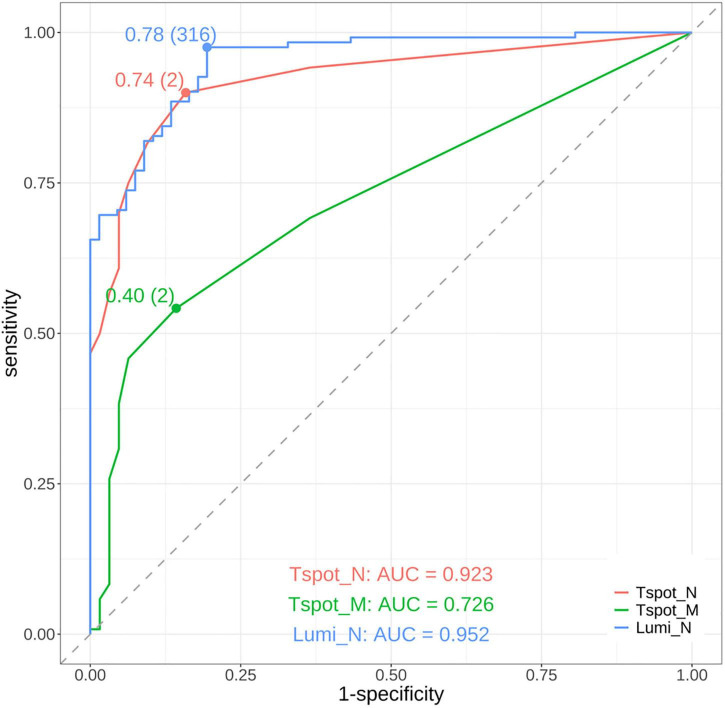
Receiver operating characteristic (ROC) curves of the assays. The red curve represents the ROC curve of the T-SPOT^®^ Discovery SARS-CoV-2 assay targeting the nucleocapsid protein (Tspot_N). The green curve represents the ROC curve of the T-SPOT^®^ Discovery SARS-CoV-2 assay targeting the membrane protein (Tspot_M). The blue curve represents the ROC curve of the antibody test using the MAGPIX^®^ system, which targets the nucleocapsid protein (Lumi_N). The area under the ROC curve (AUC) for each assay is shown in the lower part of the figure. The points on each ROC curve denote the maximum Youden index, with parentheses indicating the optimal cutoff point. On the basis of the AUC values, the Tspot_N and Lumi_N assays demonstrated good diagnostic performance and outperformed the Tspot_M assay.

**FIGURE 3:**
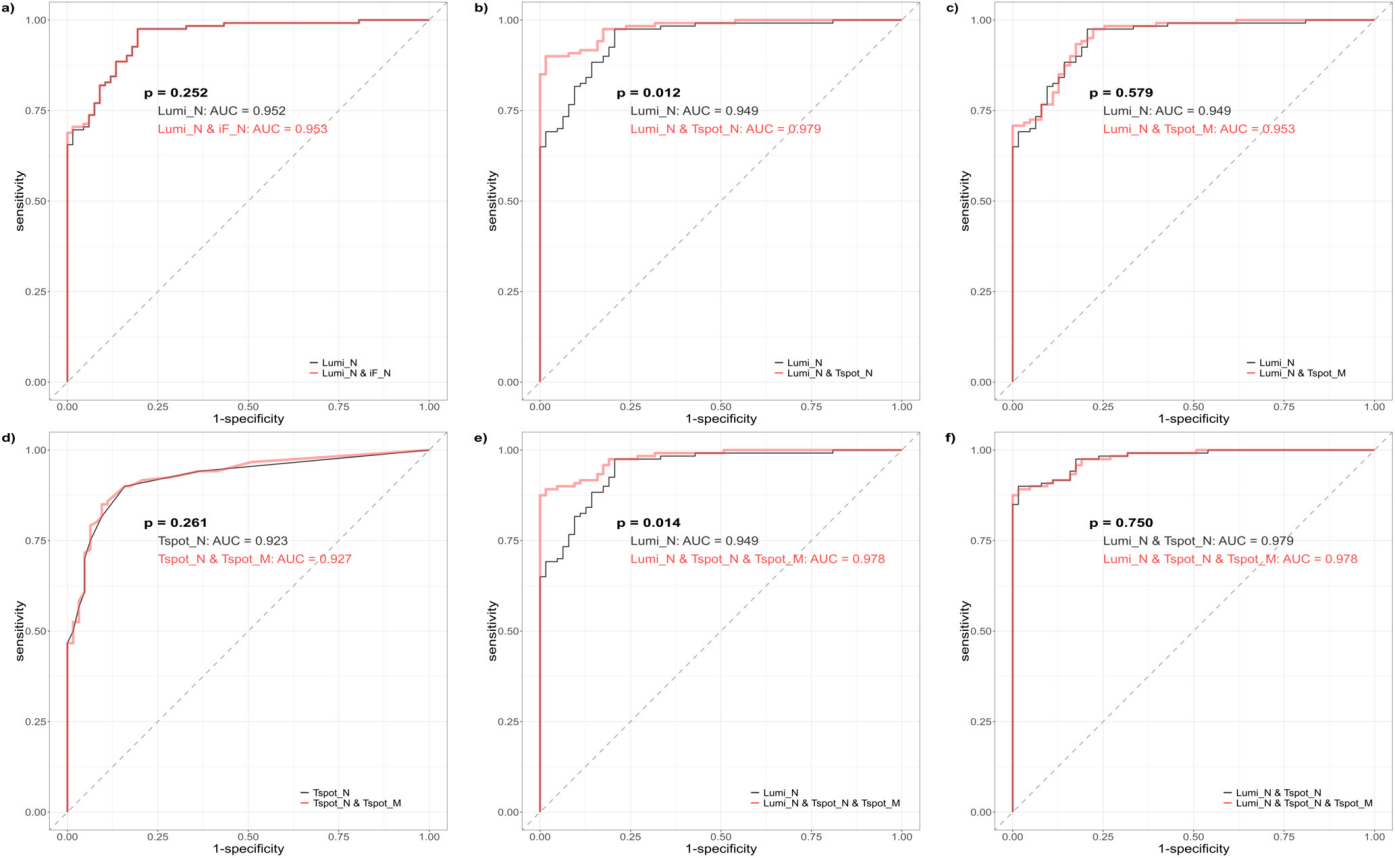
Receiver operating characteristic (ROC) curves of the combination assays. The black ROC curve represents the baseline model, which encompasses both the single and combination assays. The red ROC curve illustrates the new model that was generated by incorporating one or more assays into the baseline model. Each ROC curve was derived from logistic regression analysis. **(a)** Comparison between the Lumi_N assay (baseline model) and the combination of antibody assays Lumi_N and iF_N. **(b)** Comparison between the Lumi_N assay (baseline model) and the combination of the Lumi_N and Tspot_N assay. **(c)** Comparison between the Lumi_N assay (baseline model) and the combination of the Lumi_N and Tspot_M assay. **(d)** Comparison between the Tspot_N assay (baseline model) and the combination of the Tspot_N and Tspot_M assays. **(e)** Comparison between the Lumi_N assay (baseline model) and the combination of the Lumi_N, Tspot_N, and Tspot_M assays. **(f)** Comparison between the combination of the Lumi_N and Tspot_N assays (baseline model) and the combination of the Lumi_N, Tspot_N, and Tspot_M assays. The combination of the Lumi_N and Tspot_N assays had the highest diagnostic performance, and no additional benefit was observed when the Tspot_M assay was incorporated.

To assess the diagnostic efficacy in detecting past COVID-19 infections, we performed ROC curve analysis of the quantitative assays, specifically the Lumi_N, Tspot_N, and Tspot_M assays, and their combinations. The findings are presented in Figures 2, 3 and Supplementary Table 7. The AUC for the Tspot_M assay was 0.726, whereas the other assays and their combinations presented AUCs exceeding 0.923 (Figure 2). The use of combination assays generally increased the diagnostic performance of the individual, except those incorporating the Tspot_M assay (Figure 3). Notably, the AUC for the combination of Lumi_N and Tspot_N was significantly greater than that for the Lumi_N assay alone (*p* = 0.012 [DeLong’s test], 0.979 vs. 0.949). The combination of Lumi_N and Tspot_N achieved the highest AUC of 0.979; however, the inclusion of the Tspot_M assay slightly diminished the diagnostic performance (ΔAUC = −0.040, *p* = 0.750).

The optimal cutoff values for the Lumi_N, Tspot_N, and Tspot_M assays were established on the basis of the maximum value of the Youden index (Figure 2). The sensitivities of these optimized assays were approximately 30% higher than those assessed with the original cutoff value (Table 3), while the specificities of these assays ranged from 80.6% to 85.7%. We performed two-category and continuous NRI and IDI analyses to assess assay combinations (Table 5). Combining the Tspot_N assay with the Lumi_N assay increased the AUC from 0.949 to 0.979, with *p*-values less than 0.001 for all analyses. The Tspot_M assay showed no improved diagnostic performance when combined with other assays. The analyses confirmed that only the Lumi_N and Tspot_N combination improved COVID-19 diagnostic performance.

**TABLE 5:** Results of reclassification analyses.

Models		NRI (categorical)		NRI (continuous)		IDI	
Model_1 (baseline model)	Model_2 (new model)	Value (95% CI)	*P*-value	Value (95% CI)	*P*-value	Value (95% CI)	*P*-value
Lumi_N	Lumi_N & iF_N	−0.025 (−0.052, 0.003)	0.079	−0.284 (−0.53, −0.038)	**0.023**	−0.004 (−0.009, 0.002)	0.170
Lumi_N	Lumi_N & Tspot_N	0.180 (0.083, 0.277)	**<0.001**	0.898 (0.688, 1.108)	**<0.001**	0.168 (0.122, 0.214)	**<0.001**
Lumi_N	Lumi_N & Tspot_M	0 (−0.055, 0.055)	1.000	0.428 (0.205, 0.65)	**<0.001**	0.020 (−0.002, 0.043)	0.079
Tspot_N	Tspot_N & Tspot_M	0 NA	NA	0.274 (0.003, 0.544)	**0.047**	0.001 (−0.002, 0.004)	0.440
Lumi_N	Lumi_N & Tspot_N & Tspot_M	0.172 (0.073, 0.27)	**0.001**	0.933 (0.716, 1.15)	**<0.001**	0.170 (0.124, 0.216)	**<0.001**
Lumi_N & Tspot_N	Lumi_N & Tspot_N & Tspot_M	0.016 (−0.023, 0.054)	0.420	0.078 (−0.192, 0.349)	0.571	0.002 (−0.006, 0.01)	0.676

CI, confidence interval; NRI, net reclassification improvement; IDI, integrated discriminatory index; CI, confidence interval; Lumi_N, nucleoprotein assay of the MAGPIX^®^ system (Luminex); iF_N, iFlash-SARS-CoV-2 IgG assay; Tspot_N, nucleocapsid assay of T-SPOT^®^ Discovery SARS-CoV-2; Tspot_M, membrane protein assay of T-SPOT^®^ Discovery SARS-CoV-2. Bold values denote a *p*-value of less than 0.05.

## 4 Discussion

In this study, we assessed the efficacy of the T-SPOT assay using frozen PBMCs and their combinations with antibody tests in diagnosing a history of SARS-CoV-2 infection. Our findings indicate that the Tspot_N assay has comparable performance to the Lumi_N assay, particularly in terms of sensitivity, specificity, and AUC. Notably, the distribution of test-positive participants differed across assays, possibly due to the discrepancy between the principles underlying the functioning of the assays and targeted immunity. Specifically, IGRAs, such as the T-SPOT assay, reflect the cellular immunity mediated by T lymphocytes and their immune memory, whereas antibody tests reflect the humoral immunity associated with circulating antibodies. Numerous studies have evaluated the utility of IGRAs and their advantages over antibody tests (Barreiro et al., 2022, Fernández-González et al., 2022). IGRAs can detect COVID-19 in the earlier stages and remain positive for longer periods than antibody tests (Johnson et al., 2023). In our study, the positivity rates of the antibody assays significantly decreased in the later stages of infection. However, such differences in positivity were not observed with the IGRAs, with the Tspot_N assay even exhibiting the highest sensitivity beyond 180 days post-SARS-CoV-2 infection. T-cell assays may provide additional value in diagnosing prior COVID-19 by complementing antibody assays. As previously documented, the positivity rate of antibody assays in the PC cohort decreased over the course of COVID-19. These differences between the antibody tests and T-SPOT assays may influence the development of combination assay strategies (e.g., the combination of Lumi_N and Tspot_N).

The manufacturer’s instructions specify the use of fresh peripheral PBMCs. In surveillance studies, such as those assessing infectious disease prevalence, the use of PBMCs can be challenging owing to the complexity of their preparation. Consequently, we employed frozen and preserved PBMCs for the T-SPOT^®^ Discovery SARS-CoV-2 assay, facilitating an effective IGRA by allowing the testing of multiple samples in a single batch. A previous study compared fresh and frozen PBMCs in performing the T-SPOT COVID-19 test (Nadat et al., 2022). The results of that study revealed that frozen PBMCs were generally applicable, although some caution was warranted, and the authors emphasized the necessity of using viable PBMCs. We utilized CELLBANKER1 to store PBMCs, which ensures their viability for extended periods, maintaining a viability rate exceeding 70% at −80 °C. We prepared 2.5 × 10^6^ cells/mL viable PBMCs using a cell counter with trypan blue staining. With the Tspot_N and Tspot_M assays, interpretable results were obtained for approximately 80% of the PC cohort and 90% of the NC cohort, and the sensitivity of each assay was 62.5% and 15.3%, respectively. One study assessed the sensitivity of the T-SPOT assay using nucleocapsid protein as a stimulant (Pitiriga et al., 2023). The sensitivities in our study were expected to be slightly lower than those reported in a previous study that employed the nucleocapsid protein T-SPOT assay. The use of frozen PBMCs may have contributed to these differences in the performance of the T-SPOT nucleocapsid assays. However, as described in a previous study (Johnson et al., 2023), IFN-γ ELISPOT assays, such as T-SPOT assays, are labor intensive and require a minimum of 2 days to return results. Therefore, the use of frozen samples is essential for conducting surveillance studies using IFN-γ ELISPOT assays, particularly for prevalence surveillance studies.

Numerous studies have demonstrated the efficacy of IFN-γ release assays in identifying previous COVID-19. However, none of these studies addressed the efficacy of the IFN-γ release assay when implemented in conjunction with antibody tests. To evaluate the effects of such combination assays, we performed ROC curve, NRI, and IDI analyses, which indicated a significant improvement in the identification of prior COVID-19. Additionally, we conducted Brier score analysis as a supplementary evaluation (Vickers et al., 2019, Hilden and Gerds, 2014). The NRI and IDI primarily assess the added value of new biomarkers when implemented within existing predictive models and determine their utility in assessing the risk of clinical events (Cook, 2018). Numerous studies have evaluated the additive effects of biomarkers, such as those for acute ischemic stroke and the diagnosis of pulmonary tuberculosis (Mu et al., 2024, Ling et al., 2013), using the NRI and IDI. Importantly, the NRI, both categorical and continuous, may overestimate the improvement in diagnostic performance if the original model was poorly fit (Pepe et al., 2015). Other statistics, such as the ΔAUC and Brier score, are not affected by model quality. With respect to the combinations of the Lumi_N and Tspot_N assays, both the ΔAUC and ΔBrier scores also indicated a positive evaluation, which was consistent with the results of the NRI and IDI analyses (Supplementary Table 7). These findings support the ability to improve diagnosis with this combination assay.

IGRAs encompass several methods for detecting responses against specific proteins, such as ELISPOT (T-SPOT), ELISA detection of IFN-γ, or FCM (Johnson et al., 2023, Törnell et al., 2022). In some severe cases, T-cell counts, including those of CD4+ and CD8 + T cells, decrease. Because ELISA-based tests for detecting IFN-γ, such as the QuantiFERON SARS-CoV-2 assay, use whole blood, the results may be influenced by blood cell counts. Conversely, measuring viable T-cell counts using the T-SPOT test allows standardization of the T-SPOT assay. Moreover, ELISPOT (T-SPOT) assays exhibit advantages in sensitivity, surpassing intracellular cytokine (FCM) staining (Karlsson et al., 2003), and are up to 200 times more sensitive than ELISAs (Tanguay and Killion, 1994). Previous studies have evaluated the responses of the T-SPOT assay and other IGRAs to SARS-CoV-2 spike proteins (Jang et al., 2023, Seo et al., 2023). However, evaluations of IGRAs that target SARS-CoV-2 nucleocapsid or membrane proteins are limited. We addressed these shortcomings in this study and reported that the T-SPOT assay targeting the SARS-CoV-2 nucleocapsid protein (Tspot_N assay), alone or in combination with antibody tests, facilitated the diagnosis of past SARS-CoV-2 infection among vaccinated participants. Certain limitations of the T-SPOT assays should be acknowledged, however. The availability of interpretable test results was lower than that of antibody testing because of the presence of indeterminate and borderline results. The percentage of interpretable results of the T-SPOT assays ranged from 78.7% to 92.5%, whereas no invalid results were observed for antibody testing.

This study had several limitations. First, the participants were predominantly young, particularly within the PC cohort, with a median age of 36 years. Immune responses differ between younger and older populations, potentially influencing the outcomes of both antibody tests and IFN-γ release assays (Jo et al., 2023). Second, it is important to acknowledge occupational bias. A significant proportion of participants, 91.0% of the PC cohort and 65.7% of the NC cohort, were hospitality workers, who had been vaccinated at a high rate. Third, this study included a limited number of patients with severe infection and none with critical infection. Therefore, the relationship between disease severity and the results of the assays included in this study was not thoroughly assessed. Fourth, the number of patients in the NC cohort was relatively small, constituting approximately half of the PC cohort. Nevertheless, assuming a sample size of 67 per group, the estimated power was 89% to detect the difference between proportions of 0.88 (sensitivity of the Lumi_N and Tspot_N combination) and 0.65 (sensitivity of Lumi_N) with a two-sided α = 0.05. This finding supports the present study. Finally, similar to other studies, our study faced challenges in strictly excluding infected individuals from the negative control group, particularly those who contracted the infection within 2 weeks prior to blood collection (Goletti et al., 2021). Therefore, these results should be interpreted with caution, and additional research is needed to validate these results.

## 5 Conclusion

The use of frozen PBMCs is feasible for performing T-SPOT assays to detect a history of COVID-19, particularly when combined with antibody testing. These combinations improve diagnostic performance and may contribute to more effective prevalence studies.

## Data Availability

The original contributions presented in this study are included in this article/Supplementary material, further inquiries can be directed to the corresponding author.
