# Supplementary material for: Improving the diagnostic performance for prior COVID-19 with T-SPOT, an interferon-gamma release assay
Source: Front Microbiol. 2025 Sep 24;16:1675605. doi: 10.3389/fmicb.2025.1675605 (PMC12504223; doi:10.3389/fmicb.2025.1675605)
Supplement: Supplementary file 1 [file Data_Sheet_1.docx]

**Contents**

**Supplementary figures**

**Page 2** **Figure S1.** Histogram illustrating the frequency distribution of positive control cohorts on specific days when blood samples were collected following the most recent COVID-19 infection

**Page 3 Figure S2.** Histogram illustrating the frequency distribution of vaccinated individuals (total participants [a, n=294], positive control cohorts [b, n=114], and negative control cohorts [c, n=65]) on specific days when blood samples were collected following the most recent COVID-19 vaccination

**Supplementary tables**

**Page 4 Table S1.** Details of the vaccination status

**Page 5 Table S2.** Interpretability of T-SPOT test results

**Page 6** **Table S3.** Overview of the results of each assay

**Page 7 Table S4.** Number of positive assays in relation to the timing of blood collection

**Page 8 Table S5.** Number of positive assays in relation to the presence of symptoms

**Page 9 Table S6.** Sensitivity and specificity metrics for each period following the most recent COVID-19 infection

**Page 10 Table S7.** Results of the ΔAUC and ΔBrier analyses

**Page 2**

**
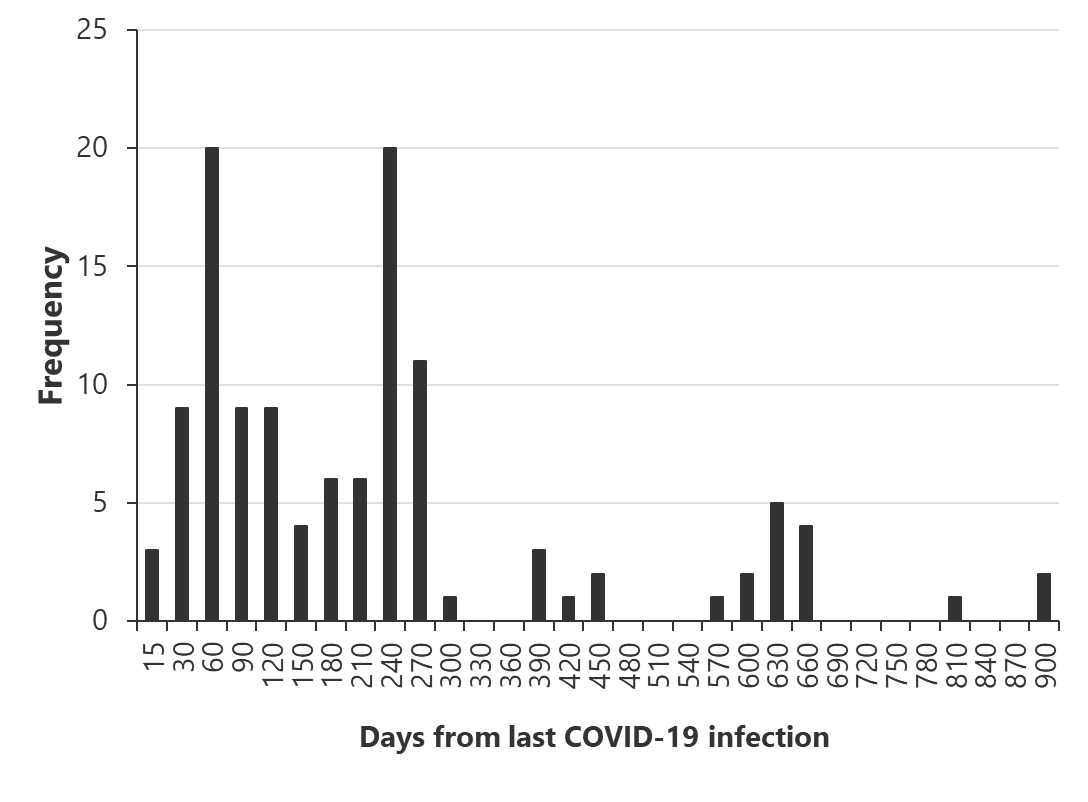
**

**Figure S1.** Histogram illustrating the frequency distribution of positive control cohorts on specific days when blood samples were collected following the most recent COVID-19 infection.

**Page 3**

**
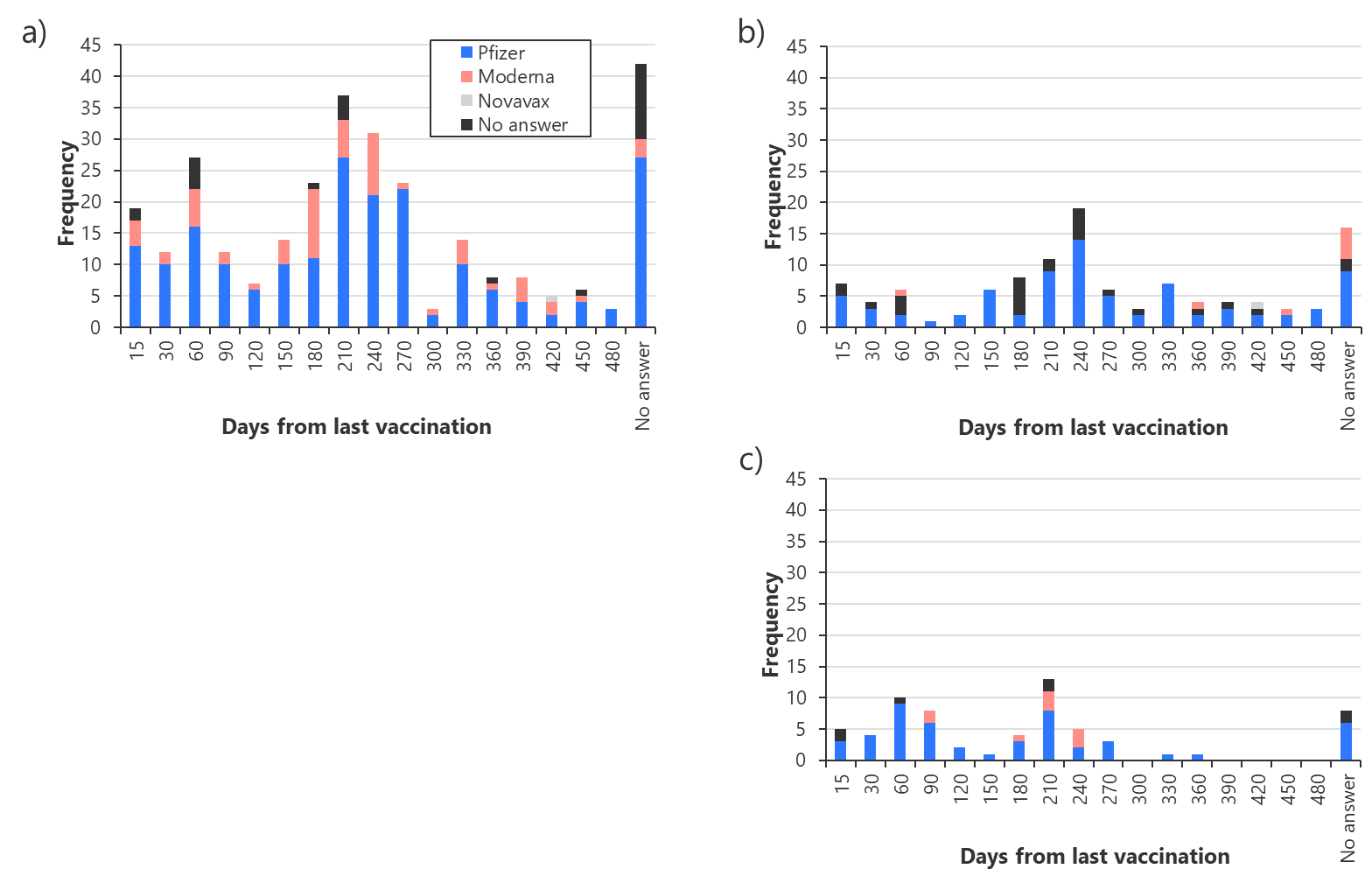
**

**Figure S2.** Histogram illustrating the frequency distribution of vaccinated individuals (total participants [a, n=294], positive control cohorts [b, n=114], and negative control cohorts [c, n=65]) on specific days when blood samples were collected following the most recent COVID-19 vaccination.

**Page 4**

**Table S1.** Details of the vaccination status

| **Cohort** | **Total** | **(n = 309)** | **PC** | **(n = 122)** | **NC** | **(n = 67)** | **PC + NC** | **(n = 189)** | **p value**  **(PC vs. NC)** |
| --- | --- | --- | --- | --- | --- | --- | --- | --- | --- |
| **Vaccination** | 294 | (95.1%) | 114 | (93.4%) | 65 | (97.0%) | 179 | (94.7%) | 0.499 |
| 4 doses (Pfizer) | 36 | (12.2%) | 8 | (7.0%) | 19 | (29.2%) | 27 | (15.1%) |  |
| 4 doses (Moderna) | 2 | (0.7%) | 0 | (0%) | 1 | (1.5%) | 1 | (0.6%) |  |
| 4 doses (Pfizer, Moderna) | 23 | (7.8%) | 6 | (5.3%) | 6 | (9.2%) | 12 | (6.7%) |  |
| 4 doses (Unknown*) | 9 | (3.1%) | 1 | (0.9%) | 5 | (7.7%) | 6 | (3.4%) |  |
| 3 doses (Pfizer) | 91 | (31.0%) | 36 | (31.6%) | 18 | (27.7%) | 54 | (30.2%) |  |
| 3 doses (Moderna) | 20 | (6.8%) | 11 | (9.6%) | 3 | (4.6%) | 14 | (7.8%) |  |
| 3 doses (Pfizer, Moderna) | 34 | (11.6%) | 12 | (10.5%) | 5 | (7.7%) | 17 | (9.5%) |  |
| 3 doses (Pfizer, Novavax) | 1 | (0.3%) | 1 | (0.9%) | 0 | (0%) | 1 | (0.6%) |  |
| 3 doses (Unknown*) | 5 | (1.7%) | 3 | (2.6%) | 1 | (1.5%) | 4 | (2.2%) |  |
| 2 doses (Pfizer) | 42 | (14.3%) | 24 | (21.1%) | 4 | (6.2%) | 28 | (15.6%) |  |
| 2 doses (Moderna) | 14 | (4.8%) | 5 | (4.4%) | 0 | (0%) | 5 | (2.8%) |  |
| 2 doses (Unknown*) | 1 | (0.3%) | 0 | (0%) | 0 | (0%) | 0 | (0%) |  |
| 1 dose (Unknown*) | 2 | (0.7%) | 2 | (1.8%) | 0 | (0%) | 2 | (1.1%) |  |
| No answer | 14 | (4.8%) | 5 | (4.4%) | 3 | (4.6%) | 8 | (4.5%) |  |

Abbreviations: PC, positive control; NC, negative control; IQR, interquartile range. Bold values denote p values less than 0.05.

* Types of vaccines were not answered.

**Page 5**

**Table S2.** Interpretability of T-SPOT test results

|  | **PC (n=122)** | | | | | | | | **NC (n=67)** | | | | | | | |
| --- | --- | --- | --- | --- | --- | --- | --- | --- | --- | --- | --- | --- | --- | --- | --- | --- |
| **Assays** | **Pos.** | **(%)** | **Bord.** | **(%)** | **Neg.** | **(%)** | **Indet.** | **(%)** | **Pos.** | **(%)** | **Bord.** | **(%)** | **Neg.** | **(%)** | **Indet.** | **(%)** |
| Tspot_N | 60 | (49.2%) | 24 | (19.7%) | 36 | (29.5%) | 2 | (1.6%) | 1 | (1.5%) | 2 | (3.0%) | 60 | (89.6%) | 4 | (6.0%) |
| Tspot_M | 15 | (12.3%) | 22 | (18.0%) | 83 | (68.0%) | 2 | (1.6%) | 2 | (3.0%) | 1 | (1.5%) | 60 | (89.6%) | 4 | (6.0%) |

Abbreviations: PC, positive control; NC, negative control; Tspot_N, nucleocapsid assay of T-SPOT^®^ Discovery SARS-CoV-2; Tspot_M, membrane protein assay of T-SPOT^®^ Discovery SARS-CoV-2; Pos. ; positive, Bord. ; borderline, Neg. ; negative, Indet. ; indeterminate.

**Page 6**

**Table S3. Overview of the results of each assay.**

|  | **PC** |  |  | **NC** |  |  |  |
| --- | --- | --- | --- | --- | --- | --- | --- |
|  | **Positivity rate (%)** | **Median** | **IQR** | **Positivity rate (%)** | **Median** | **IQR** | **p value*** |
| **iF_N** | 67.2 | - | - | 0 | - | - | - |
| **Lumi_N** | 64.8 | 2763 | 1254-7112 | 0 | 76 | 41-233 | < 0.001 |
| **Tspot_N** | 62.5 | 7 | 3.25-13.75 | 1.6 | 0 | 0-1 | < 0.001 |
| **Tspot_M** | 15.3 | 2 | 0-5.75 | 3.2 | 0 | 0-1 | < 0.001 |

Abbreviations: iF_N, iFlash-SARS-CoV-2 IgG assay; Lumi_N, nucleoprotein assay of the MAGPIX^®^ system (Luminex); Tspot_N, nucleocapsid assay of T-SPOT^®^ Discovery SARS-CoV-2; Tspot_M, membrane protein assay of T-SPOT^®^ Discovery SARS-CoV-2; IQR, interquartile range.

* Comparison between the medians of each cohort.

**Page 7**

**Table S4.** Number of positive assays in relation to the timing of blood collection

|  | **≦180^*^** | **(n=60)** |  | **>180^*^** | **(n=59)** |  |  |  |  |
| --- | --- | --- | --- | --- | --- | --- | --- | --- | --- |
|  | **Positive assays** | **(%)** | **NA** | **Positive assays** | **(%)** | **NA** | **p value** | **Odds ratio** | **(95% CI)** |
| iF_N | 52 | (86.7%) | - | 27 | (45.8%) | - | **< 0.001** | 7.56 | (2.92, 21.75) |
| Lumi_N | 53 | (88.3%) | - | 25 | (42.4%) | - | **< 0.001** | 10.1 | (3.75, 30.80) |
| Tspot_N | 27 | (61.4%) | 17 | 31 | (62.0%) | 9 | 1 | 1.03 | (0.41, 2.62) |
| Tspot_M | 7 | (15.2%) | 14 | 7 | (13.7%) | 8 | 1 | 1.13 | (0.31, 4.14) |

Abbreviations: CI, confidence interval; iF_N, iFlash-SARS-CoV-2 IgG assay; Lumi_N, nucleoprotein assay of the MAGPIX^®^ system (Luminex); Tspot_N, nucleocapsid assay of T-SPOT^®^ Discovery SARS-CoV-2; Tspot_M, membrane protein assay of T-SPOT^®^ Discovery SARS-CoV-2.

* Timing of blood collection relative to COVID-19 infection. NA is the number of participants for whom useful test results were unavailable ("test invalid" or "not interpretable [borderline]"). Bold values denote p values less than 0.05.

**Page 8**

**Table S5.** Number of positive assays in relation to the presence of symptoms

| **Assay** | **Asymptomatic (n=22)** | | | **Symptomatic (n=100)** | | |  |  |  |
| --- | --- | --- | --- | --- | --- | --- | --- | --- | --- |
|  | **Positive assays** | **(%)** | **NA** | **Positive assays** | **(%)** | **NA** | **p value** | **Odds ratio** | **(95% CI)** |
| iF_N | 17 | (77.3%) | - | 65 | (65.0%) | - | 0.323 | 0.549 | (0.146, 1.724) |
| Lumi_N | 14 | (63.6%) | - | 65 | (65.0%) | - | 1.000 | 1.061 | (0.350, 3.027) |
| Tspot_N | 9 | (40.9%) | 9 | 51 | (51.0%) | 21 | 0.289 | 1.810 | (0.565, 5.821) |
| Tspot_M | 5 | (22.7%) | 1 | 10 | (10.0%) | 21 | 0.299 | 0.468 | (0.123, 1.991) |

Abbreviations: CI, confidence interval; iF_N, iFlash-SARS-CoV-2 IgG assay; Lumi_N, nucleoprotein assay of the MAGPIX^®^ system (Luminex); Tspot_N, nucleocapsid assay of T-SPOT^®^ Discovery SARS-CoV-2; Tspot_M, membrane protein assay of T-SPOT^®^ Discovery SARS-CoV-2. NA is the number of participants for whom valid test results were unavailable ("test invalid" or "not interpretable [borderline]"). Bold values denote p values less than 0.05.

**Page 9**

**Table S6.** Sensitivity and specificity metrics for each period following the most recent COVID-19 infection

| **Lumi_N** |  |  |  |  |  |  |
| --- | --- | --- | --- | --- | --- | --- |
| **Days from infection** | **Sensitivity** | **(95% CI)** | **Specificity** | **(95% CI)** | **PC, N** | **NC, N** |
| **≦60** | 93.8% | (79.2%, 99.2%) | 100.0% | (94.6%, 100.0%) | 32 | 67 |
| **61-120** | 88.9% | (65.3%, 98.6%) | 100.0% | (94.6%, 100.0%) | 18 | 67 |
| **121-180** | 70.0% | (34.8%, 93.3%) | 100.0% | (94.6%, 100.0%) | 10 | 67 |
| **181-240** | 23.1% | (9.0%, 43.6%) | 100.0% | (94.6%, 100.0%) | 26 | 67 |
| **241-300** | 50.0% | (21.1%, 78.9%) | 100.0% | (94.6%, 100.0%) | 12 | 67 |
| **>300** | 61.9% | (38.4%, 81.9%) | 100.0% | (94.6%, 100.0%) | 21 | 67 |
| **Tspot_N** |  |  |  |  |  |  |
| **Days from infection** | **Sensitivity** | **(95% CI)** | **Specificity** | **(95% CI)** | **PC, N** | **NC, N** |
| **≦60** | 75.0% | (50.9%, 91.3%) | 98.4% | (91.2%, 100.0%) | 20 | 61 |
| **61-120** | 42.9% | (17.7%, 71.1%) | 98.4% | (91.2%, 100.0%) | 14 | 61 |
| **121-180** | 66.7% | (29.9%, 92.5%) | 98.4% | (91.2%, 100.0%) | 9 | 61 |
| **181-240** | 56.5% | (34.5%, 76.8%) | 98.4% | (91.2%, 100.0%) | 23 | 61 |
| **241-300** | 70.0% | (34.8%, 93.3%) | 98.4% | (91.2%, 100.0%) | 10 | 61 |
| **>300** | 64.7% | (38.3%, 85.8%) | 98.4% | (91.2%, 100.0%) | 17 | 61 |
| **Lumi_N & Tspot_N** |  |  |  |  |  |  |
| **Days from infection** | **Sensitivity** | **(95% CI)** | **Specificity** | **(95% CI)** | **PC, N** | **NC, N** |
| **≦60** | 100.0% | (89.1%, 100.0%) | 98.4% | (91.2%, 100.0%) | 32 | 61 |
| **61-120** | 88.9% | (65.3%, 98.6%) | 98.4% | (91.2%, 100.0%) | 18 | 61 |
| **121-180** | 100.0% | (69.2%, 100.0%) | 98.4% | (91.2%, 100.0%) | 10 | 61 |
| **181-240** | 69.6% | (47.1%, 86.8%) | 98.4% | (91.2%, 100.0%) | 23 | 61 |
| **241-300** | 81.8% | (48.2%, 97.7%) | 98.4% | (91.2%, 100.0%) | 11 | 61 |
| **>300** | 90.0% | (68.3%, 98.8%) | 98.4% | (91.2%, 100.0%) | 20 | 61 |

Abbreviations: CI, confidence interval; Tspot_N, T-SPOT^®^ Discovery using nucleocapsid protein as a stimulant; Lumi_N, antibody test targeting nucleocapsid protein using the MAGPIX^®^ system (Luminex); PC, positive control; NC, negative control.

**Page 10**

**Table S7.** Results of the ΔAUC and ΔBrier analyses

| **Models** | | **AUC** | | | **DeLong's test** | **Brier score** | | |
| --- | --- | --- | --- | --- | --- | --- | --- | --- |
| **Model_1**  **(baseline model)** | **Model_2**  **(new model)** | **Model_1** | **Model_2** | **ΔAUC (x100)** | **p value** | **Model_1** | **Model_2** | **ΔBrier (x100)** |
| Lumi_N | Lumi_N & iF_N | 0.952 | 0.953 | 0.086 | 0.252 | 0.094 | 0.095 | -0.082 |
| Lumi_N | Lumi_N & Tspot_N | 0.949 | 0.979 | 2.937 | **0.012** | 0.096 | 0.062 | 3.480 |
| Lumi_N | Lumi_N & Tspot_M | 0.949 | 0.953 | 0.384 | 0.579 | 0.096 | 0.093 | 0.384 |
| Tspo_N | Tspot_N & Tspot_M | 0.923 | 0.927 | 0.456 | 0.261 | 0.103 | 0.102 | 0.046 |
| Lumi_N | Lumi_N & Tspot_N & Tspot_M | 0.949 | 0.978 | 2.897 | **0.014** | 0.096 | 0.061 | 3.506 |
| Lumi_N & Tspot_N | Lumi_N & Tspot_N & Tspot_M | 0.979 | 0.978 | -0.040 | 0.750 | 0.062 | 0.061 | 0.026 |

Abbreviations: AUC, area under the receiver operating characteristic curve; Lumi_N, nucleoprotein assay of the MAGPIX^®^ system (Luminex); iF_N, iFlash-SARS-CoV-2 IgG assay; Tspot_N, nucleocapsid assay of T-SPOT^®^ Discovery SARS-CoV-2; Tspot_M, membrane protein assay of T-SPOT^®^ Discovery SARS-CoV-2.
